# Biopsy findings after detection of de novo donor-specific antibodies in renal transplant recipients: a single center experience

**DOI:** 10.1007/s40620-021-01040-y

**Published:** 2021-04-17

**Authors:** Christoph B. Waldecker, Panagiota Zgoura, Felix S. Seibert, Sabina Gall, Peter Schenker, Frederic Bauer, Benjamin Rohn, Richard Viebahn, Nina Babel, Timm H. Westhoff

**Affiliations:** 1 Medizinische Klinik I, Medical Department I, Marien Hospital Herne University Clinic, Ruhr-University Bochum, Hölkeskampring 40, 44625 Herne, Germany; 2 Department of Surgery, Knappschaftskrankenhaus Bochum, Ruhr-University Bochum, Herne, Germany

**Keywords:** Donor-specific antibodies, DSA, Kidney transplantation, Antibody-mediated rejection

## Abstract

**Background:**

De novo donor-specific antibodies (DSA) are associated with an increased risk of antibody-mediated rejection and a substantial reduction of allograft survival. We hypothesized that detection of DSA should prompt a biopsy even in the absence of proteinuria and loss of estimated glomerular filtration rate (eGFR). However, data on a population without proteinuria or loss of kidney function is scant, and this is the main novelty of our study design.

**Methods:**

Single center retrospective analysis on biopsy findings after detection of de novo DSA. One-hundred-thirty-two kidney and pancreas-kidney transplant recipients were included. Eighty-four of these patients (63.6%) underwent allograft biopsy. At the time of biopsy n = 50 (59.5%) had a protein/creatinine ratio (PCR) > 300 mg/g creatinine and/or a loss of eGFR ≥ 10 ml/min in the previous 12 months, whereas 40.5% did not. Diagnosis of rejection was performed according to Banff criteria.

**Results:**

Seventy-seven (91.7%) of the biopsies had signs of rejection (47.6% antibody mediated rejection (ABMR), 13.1% cellular, 20.2% combined, 10.7% borderline). Among subjects without proteinuria or loss of eGFR ≥ 10 ml/min/a (n = 34), 29 patients (85.3%) showed signs of rejection (44.1% antibody mediated (ABMR), 14.7% cellular, 11.8% combined, 14.7% borderline).

**Conclusion:**

The majority of subjects with de novo DSA have histological signs of rejection, even in the absence of proteinuria and deterioration of graft function. Thus, it appears reasonable to routinely perform an allograft biopsy after the detection of de novo DSA.

**Graphic abstract:**

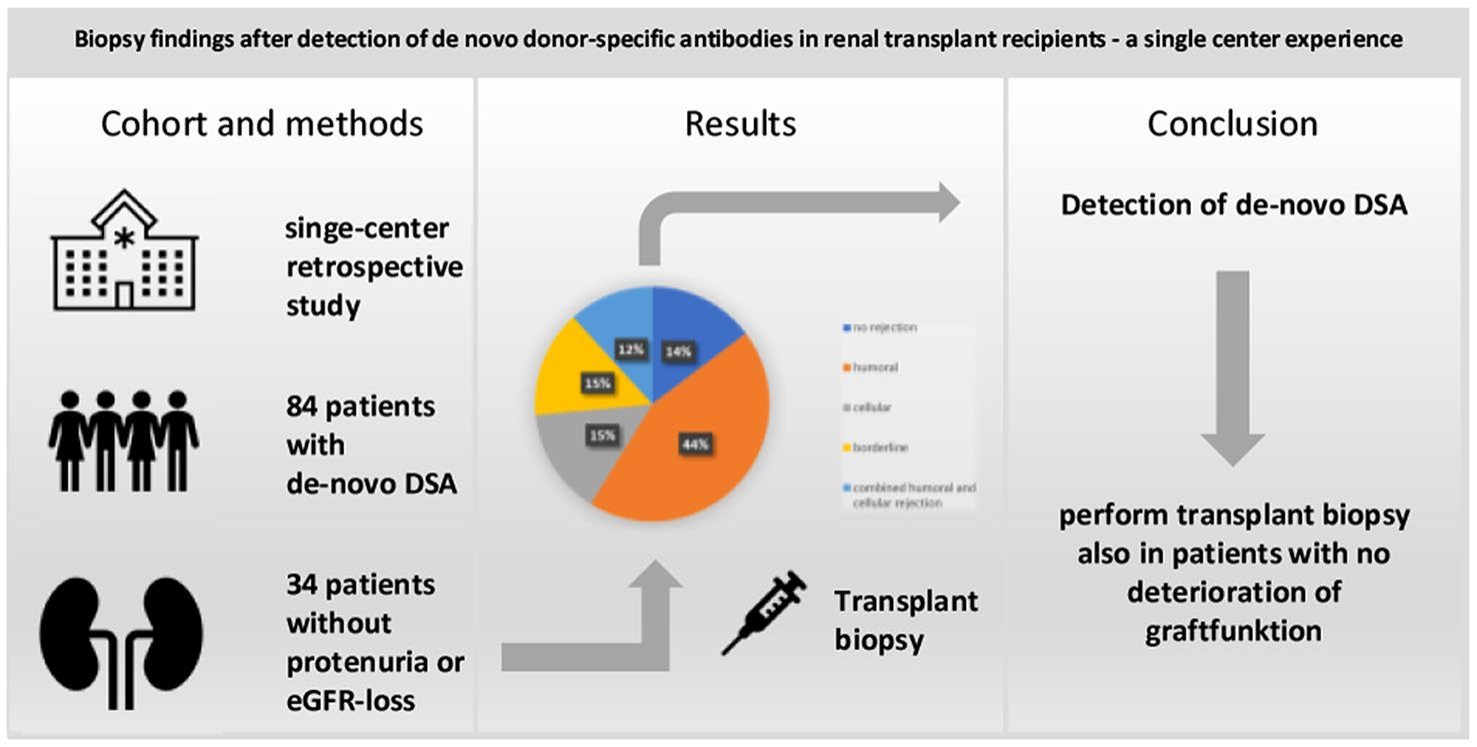

**Supplementary Information:**

The online version contains supplementary material available at 10.1007/s40620-021-01040-y.

## Introduction

Occurrence of de novo donor-specific antibodies (DSA) is associated with an increased risk of antibody-mediated rejection (ABMR) and a substantial reduction of allograft survival [1]. Five years after detection of de novo DSA 50.0% of renal transplant recipients will have returned to dialysis [2]. Therefore, an increasing number of transplant centers screen for DSA on a regular basis—e. g. every three to twelve months. It remains elusive, however, what to do in case of a positive finding regarding both potential intensification of immunosuppression and carrying out a biopsy.

**Fig. 1:**
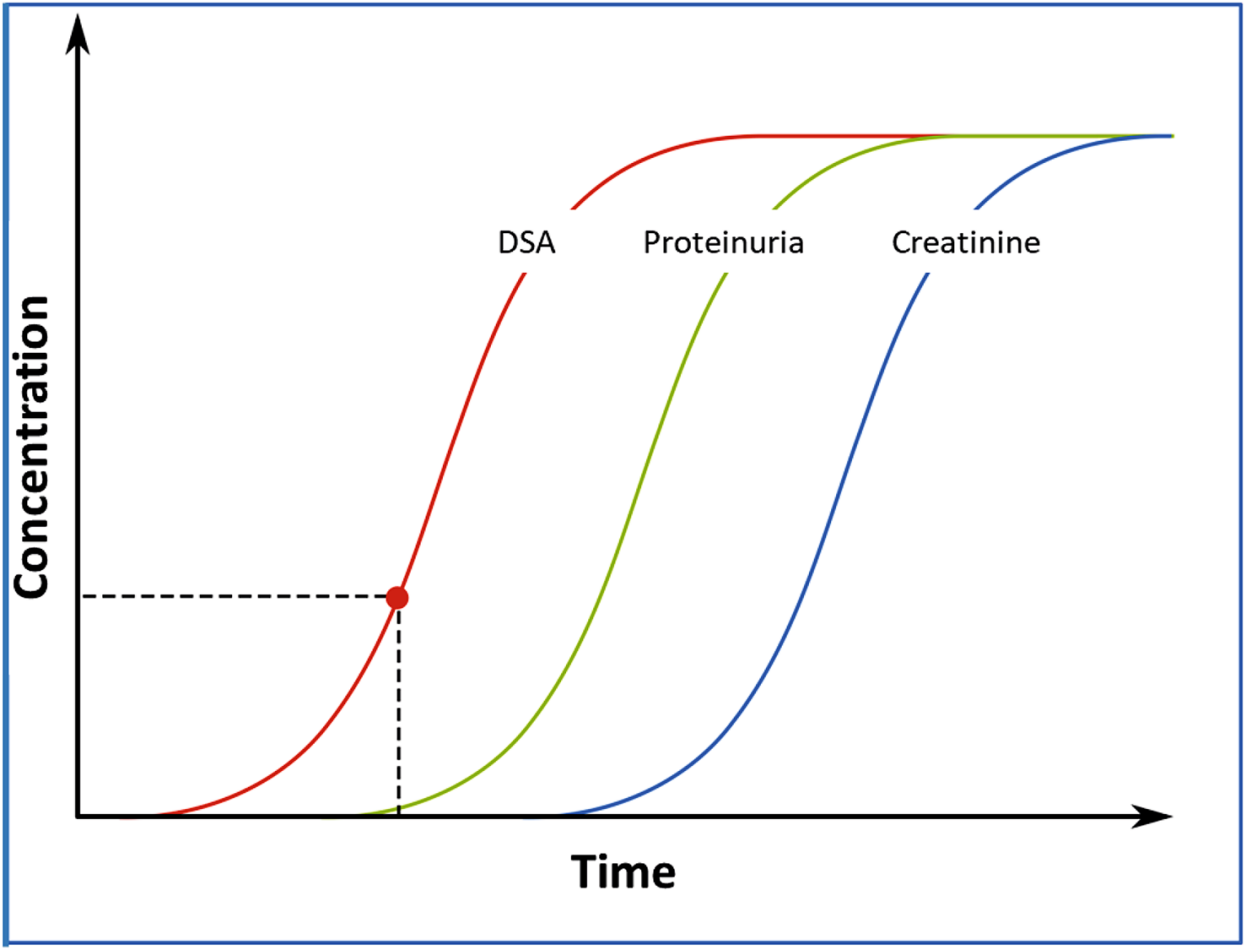
Scheme of natural course of clinical signs in antibody-mediated rejections. The red dot indicates the onset of clinically detectable proteinuria

The development of DSA constitutes the first step in the evolution of ABMR. Second, the DSA initiate inflammation with consecutive glomerular damage resulting in impaired permselectivity and proteinuria [3]. Finally, there is a deterioration of glomerular filtration resulting in a clinically detectable rise in serum creatinine concentration (Fig. 1). Treatment of ABMR is one of the biggest challenges in current transplant medicine. The more advanced the glomerular pathology, the worse the efficacy of rejection therapy. We therefore hypothesized that detection of de novo DSA should be regarded as an indication for renal allograft biopsy even in the absence of proteinuria and impaired eGFR. In 2014 we started to screen for DSA on an annual basis and changed our standard operating procedure to recommend biopsy to every transplant recipient in case of a positive finding.

There is a consensus guideline on testing and clinical management of HLA and non-HLA antibodies in transplantation, which recommends screening for DSA on a regular basis [4]. However, it describes that this decision was not unanimous and that there is a need for further research regarding “protocol biopsies at first appearance of de novo DSA to document pathologic correlation.” [4] The present study follows this research recommendation and aims to fill the gap of evidence regarding transplant recipients with DSA but without proteinuria. It describes 84 subjects after kidney or pancreas-kidney transplantation undergoing allograft biopsy after detection of de novo DSA irrespective of proteinuria and eGFR.

## Methods

### Study design and protocol

We performed a retrospective single center analysis including all renal transplant recipients with detection of de novo DSA at the transplant center of Ruhr University Bochum, Germany, between 2014 and 2018. Anti-HLA DSA are routinely screened once a year in our transplant center. Starting in 2016, patients were advised to undergo biopsy in case of de novo DSA irrespective of proteinuria or loss of eGFR. Analyses were performed using the Luminex^R^ technology [5]. All anti-HLA antibodies were tested for donor-specificity and mean fluorescence intensity (MFI) levels. The lowest antibody concentration in this study was 500 MFI. Patients who tested positive for de novo DSA were encouraged to undergo biopsy of the renal allograft regardless of proteinuria and eGFR slope. The present work examines the histological findings of these biopsies including electron microscopy results, and describes the proportion of subjects with acute or chronic antibody-mediated rejection, cellular rejection, or a combination of both entities. In order to elucidate whether performing a biopsy is clinically conducive even in the absence of proteinuria, these subjects were analyzed in a predefined subgroup analysis.

Most of the biopsy specimens (89.3%) were analyzed by the same experienced histopathological institute. Diagnosis of cellular and ABMR was performed in accordance with 2013/2017 Banff criteria [6, 7]. Acute and chronic humoral rejections were summarized as “ABMR”. The center’s standard immunosuppressive regimen consisted of a calcineurin inhibitor (CNI; tacrolimus or cyclosporine), mycophenolic acid, prednisolone and induction therapy with either basiliximab or thymoglobulin.

### Statistical analysis

Numeric data are presented as mean ± standard deviation or median and IQR. Data were tested for normal distribution by the Kolmogorov–Smirnov test. Numeric data of subjects with proteinuria and/or loss of eGFR were compared to subjects without proteinuria or loss of eGFR by Student’s t-test in case of normal distribution, otherwise by Mann–Whitney U test. Comparison of categorical parameters was performed by Pearson-Chi^2^-test. A logistic regression model was used to define the association of age, MFI of DSA, HLA class I/II antibodies, and time until detection of de novo DSA on the diagnosis of rejection in allograft biopsy analysis. p < 0.05 was regarded statistically significant. All statistical analyses were performed using SPSS Statistics 26 (SPSS Inc, Chicago, Illinois, USA) and Prism 5 (GraphPad Software, La Jolla, California, USA).

## Results

In the period between 1997 and 2018, 1,878 patients received a kidney only or pancreas-kidney transplant in our center. Between 2014 and 2018 a total of 865 patients underwent screening for DSA. De novo DSA were detected in 132 (15.3%) renal or pancreas/kidney transplant recipients. One hundred twenty-eight (97.0%) of these subjects had an MFI level > 500. Eighty-four (63.6%) of the patients with de novo DSA agreed to undergo allograft biopsy irrespective of proteinuria and course of eGFR. Fifty-three (63.1%) were kidney only recipients, 36.9% (n = 31) were pancreas-kidney recipients. Among those with kidney only transplantation, the majority of subjects were transplanted after postmortal donation (66.0%), a minority (34.0%) after living donation. Among patients undergoing biopsy, 84.5% had their first renal allograft, 15.5% underwent two or more transplantations. At the time of de novo DSA detection, mean age of the transplant recipients undergoing biopsy was 52 years (IQR 44.8–57.0). The lowest antibody level of a patient having biopsy and proven ABMR was 999 MFI. Table 1 provides an overview on epidemiology, transplant data, and anti-HLA DSA findings. Table 2 provides data on the origin of end-stage renal disease (ESRD) and immunosuppression.

**Table 1:** Epidemiological and transplant related data of the study population

	Overall study population	Subjects undergoing biopsy	Subjects not undergoing biopsy	P	Subjects undergoing biopsy with proteinuria > 300 mg/g creatinine and/or eGFR loss ≥ 10 ml/min in the past 12 months	Subjects undergoing biopsy with proteinuria ≤ 300 mg/g Creatinine and eGFR loss < 10 ml/min in the past 12 months	P
General characteristics n, (%)	132	84 (63.6%)	48 (36.4%)		50 (59.5%)	34 (40.5%)	
Female gender n, (%)	60 (45.5%)	32 (38.1%)	28 (58.3%)	0.025	19 (38.0%)	13 (38.2%)	0.983
Age at time of transplantation in years(median, IQR)	47.5 38.0–55.25	46 38.0–53.25	50 38.0–60.0	0.289	44 37.0–52.0	50 43.25–55.75	0.043
Time on dialysis in months (median, IQR)	46 16.0–89.0	44 16.0–89.0	47 22.0–89.5	0.99	43 15.75–93.0	51 16.0–78.0	0.916
Transplant characteristics							
Kidney only transplantation n, (%)	84 (63.6%)	53 (63.1%)	31 (64.6%)	0.895	30 (60.0%)	23 (67.6%)	0.318
Pancreas-Kidney Transplanation n, (%)	48 (36.4%)	31 (36.9%)	17 (35.4%)	0.895	20 (40.0%)	11 (32.4%)	0.318
Live donor transplantation n, (%)	22 (26.2%)	18 (33.9%)	4 (12.9%)	0.052	10 (20.0%)	8 (23.5%)	0.699
First renal transplant n, (%)	112 (84.9%)	71 (84.5%)	41 (85.1%)	0.889	43 (86.0%)	28 (82.4%)	0.642
HLA-mismatch – mean ± SD A B DR	1.12 ± 0.65 1.31 ± 0.66 1.31 ± 0.72	1.17 ± 0.67 1.28 ± 0.68 1.35 ± 0.69	1.03 ± 0.61 1.38 ± 0.62 1.23 ± 0.76	0.384 0.674 0.586	1.22 ± 0.59 1.27 ± 0.71 1.38 ± 0.61	1.09 ± 0.75 1.30 ± 0.63 1.30 ± 0.80	0.131 0.602 0.061
DSA HLA Class I n, (%)	45 (34.1%)	23 (27.4%)	22 (45.8%)	0.717	11 (22.0%)	12 (35.3%)	0.285
DSA HLA Class II n, (%)	66 (50.0%)	43 (51.2%)	23 (47.9%)	0.031	28 (56.0%)	15 (44.1%)	0.185
DSA HLA Class I and II n, (%)	21 (15.9%)	18 (21.4%)	3 (6.25%)	0.022	11 (22.0%)	7 (20.6%)	0.877
MFI (mean ± SD)	8,478 ± 7,276	10,283 ± 7,339	5,394 ± 6,026	< 0.001	10,941 ± 7,335	9,306 ± 7,235	0.258
Time since transplant at DSA detection (months; median, IQR)	55.5 12.0–100.25	44 3.0–94.25	61 36.75–123.0	0.016	63.5 3.75–119.25	27 3.25–71.0	0.073
Time between detection of DSA and biopsy (months; median, IQR)	1.0 0.0–12.5	1.0 0.0–12.5	–	–	1.0 0.0–13.0	2.0 0.0–11.0	0.370
eGFR (ml/min) at time of biopsy (mean ± SD)	35.6 ± 18.5	31.7 ± 16.8	42 ± 19.3	0.001	25.9 ± 14.2	39.3 ± 16.9	< 0.001
Proteinuria at time of biopsy (mg/g Creatinine, mean ± SD)	510 ± 1,360	540 ± 850	440 ± 1,950	< 0.001	860 ± 1,040	160 ± 110	< 0.001
eGFR loss (ml/min) within 12 months before biopsy(mean ± SD)	4.9 ± 9.3	7.0 ± 11.0	1.3 ± 2.9	0.002	11.8 ± 12.6	0.8 ± 2.1	< 0.001

**Table 2:** Immunosuppression and cause of end-stage renal disease

Immunosuppression at time of biopsy	Overall study population (n = 132)	Subjects undergoing biopsy (n = 84)	Subjects not undergoing biopsy (n = 48)	P	Subjects undergoing biopsy with proteinuria > 300 mg/g creatinine and/or eGFR loss ≥ 10 ml/min in the past 12 months (n = 50)	Subjects undergoing biopsy with proteinuria ≤ 300 mg/g creatinine and eGFR loss < 10 ml/min in the past 12 months (n = 34)	P
Triple immunosuppression	113 (85.6%)	74 (89.2%)	39 (81.3%)	0.142	42 (84.0%)	32 (94.1%)	0.320
Mono/dual immunosuppression	17 (12.9%)	8 (9.5%)	9 (18.4%)	0.142	6 (12.0%)	2 (5.9%)	0.320
Steroids	124 (93.9%)	81 (96.4%)	43 (89.6%)	0.042	47 (94.0%)	34 (100%)	0.397
Azathioprin	8 (6.1%)	7 (8.5%)	1 (2.1%)	0.140	4 (8.0%)	3 (8.8%)	0.928
Mycophenolic acid	108 (81.8%)	67 (79.8%)	41 (85.4%)	0.586	38 (76.0%)	29 (85.3%)	0.480
Cyclosporine	28 (21.2%)	21 (25.0%)	7 (14.6%)	0.075	12 (24.0%)	9 (26.5%)	0.881
Tacrolimus	86 (65.2%)	56 (66.6%)	32 (66.7%)	0.664	32 (64.0%)	24 (70.6%)	0.707
mTOR inhibitors	13 (9.9%)	6 (7.1%)	7 (14.6%)	0.183	5 (10.0%)	1 (2.9%)	0.200
Cause of end-stage renal disease							
Nephrosclerosis	11 (8.3%)	9 (10.7%)	2 (4.2%)	0.513	4 (8.0%)	5 (14.7%)	0.564
Glomerulonephritis	33 (25.0%)	21 (25.0%)	12 (25.0%)	0.792	13 (26.0%)	8 (23.5%)	0.797
Polycystic kidney disease	9 (6.8%)	4 (4.8%)	5 (5.9%)	0.408	2 (4.0%)	2 (5.9%)	0.691
Interstitial nephritis	3 (2.3%)	3 (3.6%)	0 (0.0%)	0.185	2 (4.0%)	1 (2.9%)	0.797
Diabetic nephropathy	48 (36.4%)	30 (35.7%)	18 (37.5%)	0.864	19 (38.0%)	11 (32.4%)	0.476
Alport’s Syndrome	5 (3.8%)	3 (3.6%)	2 (4.2%)	0.863	1 (2.0%)	2 (5.9%)	0.347
Other	15 (11.4%)	9 (10.7%)	6 (12.5%)	0.756	6 (12.0%)	3 (8.8%)	0.797
Unknown	8 (6.1%)	5 (5.9%)	3 (6.3%)	0.660	3 (6.0%)	2 (5.9%)	0.982

Thirty-four of 84 patients (40.5%) underwent biopsy without significant eGFR loss and/or proteinuria as defined above. Fifty (59.5%) of the patients undergoing biopsy had had an eGFR loss ≥ 10 ml/min 12 months prior to biopsy and/or proteinuria > 300 mg/g creatinine.

In subjects undergoing biopsy, de novo DSA were detected after a median of 44 months (IQR 3.0–94.25) post-transplant. The time between transplantation and detection of de novo DSA tended to be higher in subjects with proteinuria and/or deterioration of allograft function (67.5 vs. 27.5, p 0.073, Table 1), whereas the time between detection of DSA and biopsy was comparable (1.0 vs. 2.0, p 0.370, Table 1). Twenty-three (27.4%) of these antibodies corresponded to HLA class I, 51.2% (n = 43) to class II, 21.4% (n = 18) to a combination of class I and II. MFI levels ranged from 700 to 27,800 with a mean of 10,283 ± 7,339. MFI values were unavailable for 1 individual. At the time of biopsy, mean proteinuria was 540 ± 850 mg/g creatinine, mean eGFR was 31.7 ± 16.8 ml/min and mean decline of eGFR in the last 12 months was 7.0 ± 11.0 ml/min. Whereas data on quantified proteinuria at the time of biopsy were unavailable for 8 patients, and the 12-month course of eGFR was missing for 5 of the subjects undergoing indicated biopsy, the data set was complete for subjects undergoing biopsy without proteinuria or deterioration of eGFR.

The time until DSA detection differed significantly between patients, who agreed to undergo biopsy (44 months, IQR 3.0–94.25) vs. those who did not (61 months, IQR 36.75–123.0, p = 0.016). Moreover, these groups differed in mean MFI levels (10,283 ± 7,339 vs. 5,394 ± 6,026, p < 0.001), eGFR at time of biopsy (31.7 ± 16.8 vs. 42 ± 19.3 ml/min, p = 0.001), proteinuria at the time of biopsy (540 ± 850 vs. 440 ± 1950 mg/g creatinine) and the loss of eGFR in the year prior to the biopsy (7.0 ± 11.0 vs. 1.3 ± 2.9 ml/min, p = 0.002; Table 1).

**Fig. 2:**
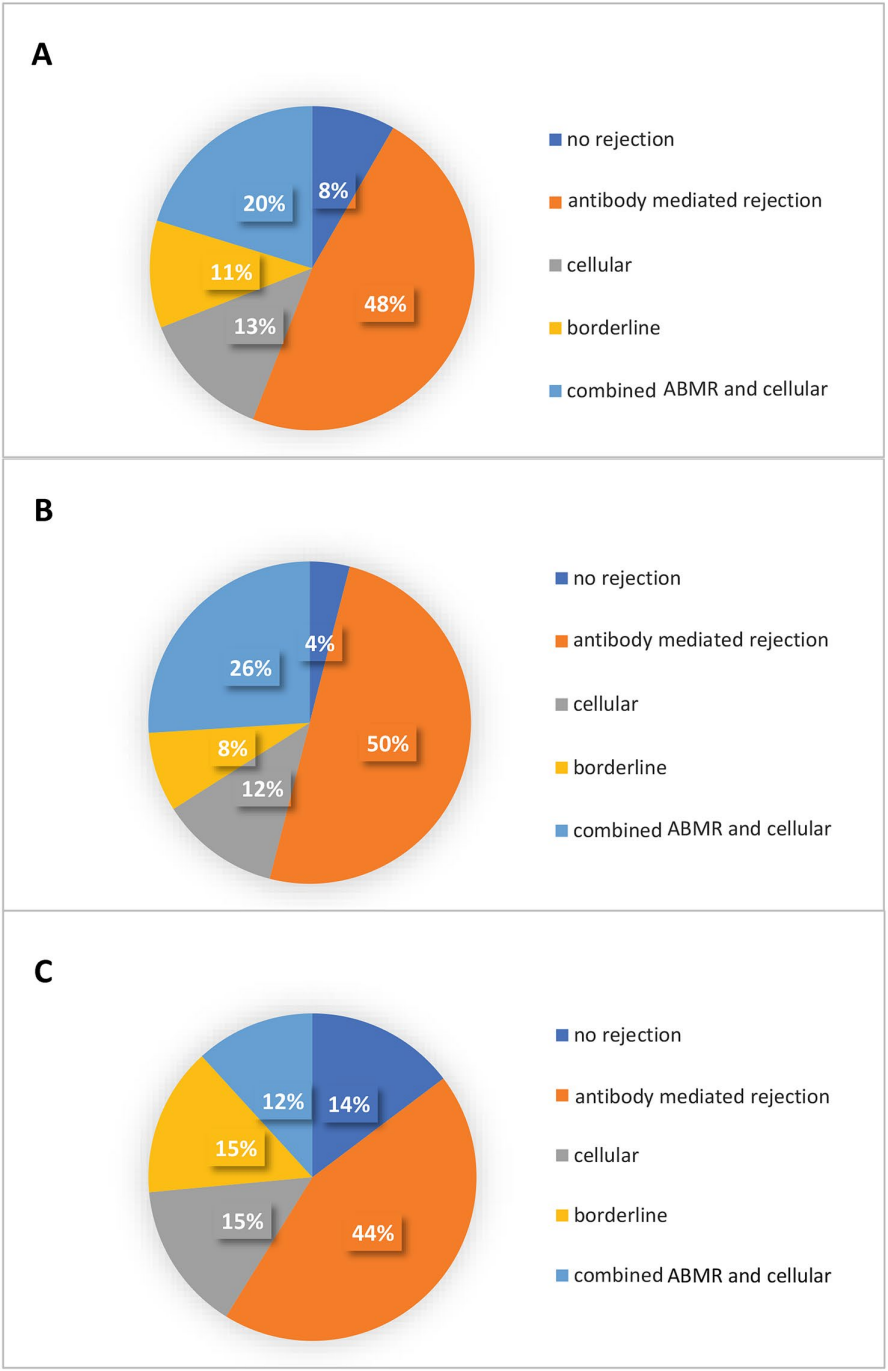
Biopsy findings in subjects undergoing biopsy after detection of de novo donor-specific antibodies (DSA). The figure describes the proportion of subjects with antibody-mediated rejections (ABMR), cellular rejections, borderline rejections, a combination of ABMR and cellular rejections, or exclusion of rejection in **a** the overall population with de novo DSA (n = 84), **b** those with proteinuria > 300 mg/g creatinine and/or loss of eGFR ≥ 10 ml/min in the previous 12 months (n = 50), and **c** those with proteinuria < 300 mg/g creatinine and loss of eGFR < 10 ml/min (n = 34)

Seventy-seven of the biopsies resulted in a diagnosis of rejection, thereby, corresponding to 91.7% of the study population. There was no significant difference in rejections between the population of patients that had combined pancreas/kidney transplantation and kidney only transplantation (p = 0.971). Among patients in whom rejection occurred, ABMR criteria were met in n = 40 (47.6%) of them. Cellular rejection was found in n = 11 (13.1%), and n = 17 patients (20.2%) showed signs of both ABMR and cellular rejection. Borderline rejection was found in n = 9 patients (10.7%), while 7 patients (8.3%) had no rejection. Among subjects undergoing biopsy without proteinuria > 300 mg/g creatinine or loss of eGFR ≥ 10 ml/a (n = 34), 85.3% (n = 29) had biopsy-proven rejection (44.1% ABMR, 14.7% cellular rejection, 11.8% combined, 14.7% borderline, 14.7% no rejection). Among subjects with proteinuria and/or deterioration of eGFR, n = 48 (96.0%) had biopsy-proven rejection (50.0% ABMR, 17.7% cellular, 38.2% combined, 8.0% borderline, 4.0% no rejection). Electron microscopy was performed in 26 cases. Thirty point seven% (n = 8) showed signs of chronic transplant glomerulopathy. There was no difference in frequency of peritubular capillary basement membrane multilayering between the group with proteinuria and/or eGFR loss and the group without proteinuria and eGFR-loss (p = 0.393). The findings are summarized in Fig. 2 and Table 3. Banff lesion scores are presented in Supplemental Table 1 [8]. The prevalence of biopsy-proven rejection tended to be higher in those with proteinuria/deterioration of graft function (p = 0.08).

**Table 3:** Biopsy findings on rejection in transplant recipients after detection of de novo donor- specific antibodies (DSA)

Kind of rejection	All biopsies n = 84	Patients with eGFR loss ≥ 10 ml/a and/or proteinuria > 300 mg/g creatinine n = 50	Patients with eGFR loss < 10 ml/a and proteinuria < 300 mg/g creatinine n = 34	p
Borderline n, (%)	9 (10.7%)	4 (8.0%)	5 (14.7%)	0.329
ABMR n, (%) - acute - chronic - combined	40 (47.6%) 26 (65.0%) 6 (15.0%) 8 (20.0%)	25 (50.0%) 15 (60.0%) 3 (12.0%) 7 (28.0%)	15 (44.1%) 11 (73.3%) 3 (20.0%) 1 (6.7%)	0.596 0.237 0.602 0.066
Cellular n, (%) - Banff 1a - Banff 1b - Banff 2	11 (13.1%) 6 (54.55%) 3 (27.27%) 2 (18.18%)	6 (17.7%) 5 (83.3%) 1 (16.7%) 0 (0.0%)	5 (14.7%) 1 (20.0%) 2 (40.0%) 2 (40.0%)	0.718 0.066 0.201 0.039
Combined cellular and ABMR	17 (20.2%)	13 (38.2%)	4 (11.8%)	0.111
No rejection n, (%)	7 (8.3%)	2 (4.0%)	5 (14.7%)	0.175

*ABMR *antibody-mediated rejection

In the logistic regression model, neither age, MFI of DSA, HLA class I/II antibodies, nor time until detection of de novo DSA predicted the overall rejections or ABMR (p > 0.05 each; Table 4).

**Table 4:** Logistic regression model on the association of age, MFI of donor-specific antibodies (DSA), HLA class I/II antibodies, and time until detection of de novo DSA with the diagnosis of rejection (overall) and antibody-mediated rejection (ABMR) in allograft biopsy analysis

N = 84	HR (95%CI) for detection of any kind of rejection	P	HR (95%CI) for detection of ABMR	p
Age at time of transplantation in years	0.996 (0.919–1.080)	0.926	1.045 (0.996–1.096)	0.070
Time since transplant at DSA	1.006 (0.989–1.023)	0.480	1.001 (0.993–1.010)	0.779
MFI	1.000 (1.000–1.000)	0.297	1.000 (1.000–1.000)	0.068
HLA I	0.965 (0.077–12.113)	0.978	0.676 (0.202–2.261)	0.525
HLA II	2.556 (0.223–29.267)	0.450	1.812 (0.436–7.530)	0.413

## Discussion

Nowadays, screening for DSA is performed on a regular basis by many but not all transplant centers. The present data strongly support this approach. Moreover, they provide implications for the diagnostic approach after detection of de novo DSA. The majority of patients in this cohort showed histological signs of rejection irrespective of proteinuria or loss of allograft function. Interestingly, the histological findings did not only present signs of ABMR but also of rejections mediated by cellular immunity.

Reports from the last three decades consistently describe the crucial role of allograft biopsies to guide immunosuppressive medication after renal transplantation. Historical reports demonstrate that indication biopsies (biopsies due to proteinuria or deterioration of eGFR) are associated with change in immunosuppression in approximately 40.0% of the patients [9]. In contrast to indication biopsies, (surveillance) intend to detect subclinical renal pathologies including rejections, viral nephritides, recurrence of glomerular diseases, and calcineurin inhibitor-induced tissue damage. A potential prognostic benefit of performing biopsies in these cases is less well established than by indication biopsies. However, biopsies in subclinical patients doubtlessly provide prognostic information, e. g. by quantification of interstitial fibrosis and tubular injury (IF/TA) [10, 11]. The proportion of patients with a subsequent change of immunosuppressive medication is necessarily lower than in indication biopsies. The present findings show that de novo DSA constitute a valuable biomarker that selects renal transplant recipients who might benefit from a biopsy despite not having proteinuria or impaired allograft function. The presence of de novo DSA indicates a highly increased probability of pathological allograft histology with therapeutic implications.

In a retrospective French study, subclinical ABMR was detected in 41.0% of biopsies following the detection of de novo DSA [12]. These data necessitate confirmation by other transplant centers. Our data are very much in line with the findings from France: 44.0% had a histological diagnosis of ABMR without proteinuria or impairment of allograft dysfunction. In the French study, stable allograft function was defined by eGFR and covered a period of three months. Proteinuria, however, was not defined as an exclusion criterion. Our data expand the implications of the French results to renal transplant recipients with stable renal allograft function in the previous 12 months and to those without proteinuria. Thus, the present findings show that biopsy is decisive even in the absence of proteinuria, which usually precedes deterioration of GFR. A study from Wisconsin reports the histological results of 29 renal transplant recipients with DSA and stable allograft function with comparable results [13]. However, only new-onset proteinuria was defined as an exclusion criterion, whereas preexisting proteinuria was not. Interestingly, the proportion of patients with rejections was only slightly higher among subjects with proteinuria and/or loss of eGFR. ABMR occurred in 50.0% of patients with allograft dysfunction and in 44.0% without proteinuria and/or loss of eGFR.

A study from the United States reports 54 transplant patients with de novo DSA and demonstrates that the dnDSA class and sum MFI at baseline appear to be prognostic [14]. Moreover, it shows that patients without ABMR at time of detection may benefit from a follow-up biopsy within one year. It does not describe, however, a population without proteinuria.

Figure 1 schematically illustrates the natural course of ABMR. The occurrence of de novo DSA usually precedes the onset of proteinuria, which in turn precedes a deterioration of eGFR. The prognosis of ABMR, however, crucially depends on the time of diagnosis. The later the diagnosis, the more irreversible the damage in renal tissue. The present approach of performing a biopsy prior to the onset of proteinuria and allograft dysfunction may therefore improve the outcome of these patients. To this end, the interval of DSA screening may be critically discussed. As mentioned above, the interval was 12 months in the present cohort. De novo DSA frequently develop after the first month post-transplant with an average time of onset of 4–5 years [15–17]. With regard to the high percentage of patients with rejections, it may be wise to shorten the DSA screening interval in the early period after transplantation. Six patients had MFI levels of 500–999, of whom two had had a transplant biopsy. None of these patients developed histological signs of rejection indicating a potentially lower immunological risk with very low DSA concentrations. This finding is in line with current recommendations to regard an MFI of 1000 as the cut-off for further diagnostic investigations [18].

The time between transplantation and detection of DSA tended to be higher in subjects with proteinuria and/or deterioration of GFR, whereas there was no difference in the time between detection of DSA and biopsy. Thus, a delay in biopsy cannot explain the finding that histological signs of rejections occur in a comparable frequency in subjects with and without an impairment of allograft function.

Interestingly, several patients did not show signs of ABMR but of acute cellular rejections. This finding is somewhat surprising with regard to the median time of 57 months after transplantation. Acute cellular rejections usually occur in the first year post-transplant. However, a high coincidence of both entities was reported in heart transplant recipients as well [19]. De novo DSA may be regarded as a biomarker of “underimmunosuppression”, which not only increases the risk of ABMR but also of cellular rejections. In our study population the coincidence of signs of cellular and antibody-mediated rejections occurred in 8.0% of the overall study population and in 14.0% of subjects without proteinuria and allograft dysfunction.

Fifteen% of ABMR met the histological signs of chronicity, whereas 65.0% were regarded as acute (20.0% combined). Concerning cellular rejections, the most prevalent Banff stage after borderline-rejection was Ia. Neither acute or chronic antibody-mediated rejections, nor the individual categories of cellular rejection occurred significantly more often in subjects with impaired allograft function than in those without it. Thus, the findings on individual rejection categories widely correspond to the overall findings in this population. In patients with subclinical ABMR, the timespan between transplantation and DSA detection was far shorter than in those with graft dysfunction. This finding is likely to be explained by the fact that screening for DSA was introduced in the routine post-transplant management in this center in 2014. Thus, patients who underwent transplant before 2014 had a higher probability of developing graft dysfunction prior to detection of DSA.

The logistic regression model did not detect a predictive value of age, MFI, HLA class I/II antibodies, and time until detection of de novo DSA on the diagnosis of rejection in allograft biopsy. Thus, obtaining a biopsy may be wise in all patients with de novo DSA and not only in a subgroup of them. HLA class II mismatch and younger age (e.g. due to noncompliance) have been described as risk factors for ABMR [20]. These are actually risk factors for the genesis of de novo DSA. Once DSA have been established—like in the present study population—the risk of developing ABMR is obviously independent of these parameters.

Our study is limited by its single-center character and the study size. To the best of our knowledge, however, it constitutes the first analysis of biopsy findings in renal transplant recipients with de novo DSA who have neither deterioration of allograft function, nor proteinuria. Moreover, the definition of < 10 ml eGFR loss in the previous 12 months as “stable renal allografts” encompasses patients with milder allograft dysfunctions. We decided for this value, however, due to the high frequency of unspecific prerenal changes in serum creatinine concentrations after kidney transplantation.

In conclusion, the present study shows that the majority of patients with de novo DSA present signs of rejection in allograft biopsies, even in the absence of proteinuria or eGFR loss. Since the prognosis of rejection therapy crucially depends on early diagnosis, it appears reasonable to perform an allograft biopsy after the detection of de novo DSA, irrespective of proteinuria or eGFR loss.

## Supplementary Information

Below is the link to the electronic supplementary material.

Supplementary file1 (DOCX 17 KB)

## Data Availability

Software application (SPSS, Excel).

## Abbreviations

ABMR: Antibody-mediated rejection
DSA: Donor-specific antibodies
eGFR: Estimated glomerular filtration rate
HLA: Human leukocyte antigen
IQR: Interquartile range
MFI: Median fluorescence intensity
PCR: Proteinuria creatinine ratio

## Declarations

### Availability for data and material

All data generated or analyzed during this study are included in this published article and its supplementary information files.
