# Supplementary material for: Biopsy findings after detection of de novo donor-specific antibodies in renal transplant recipients: a single center experience
Source: J Nephrol. 2021 Apr 17;34(6):2017–26. doi: 10.1007/s40620-021-01040-y (PMC8610940; doi:10.1007/s40620-021-01040-y)
Supplement: Supplementary file 1 — Supplementary file1 (DOCX 17 KB) [file 40620_2021_1040_MOESM1_ESM.docx]

**Supplement Table 1: Banff lesion scores**

|  | **All biopsies**  **n=84** | **Patients with eGFR loss ≥ 10 ml/a and/or proteinuria > 300 mg/g creatinine**  **n=50** | **Patients with eGFR loss < 10ml/a and proteinuria < 300 mg/g creatinine**  **n=34** | **p** |
| --- | --- | --- | --- | --- |
| **i>0**  **n, (%)** | 45  (53.6%) | 31  (62.0%) | 14  (41.2%) | 0.059 |
| **t>0**  **n, (%)** | 45  (53.6%) | 29  (58.0%) | 16  (47.1%) | 0.275 |
| **ptc>0**  **n, (%)** | 38  (45.2%) | 26  (52.0%) | 12  (35.3%) | 0.096 |
| **g>0**  **n, (%)** | 24  (28.6%) | 15  (30.0%) | 9  (26.5%) | 0.693 |
| **ptc+g≥2**  **n, (%)** | 28  (33.3%) | 19  (38.0%) | 9  (26.5%) | 0.198 |
| **c4d=1**  **n, (%)** | 28  (33.3%) | 19  (38.0%) | 9  (26.5%) | 0.354 |
| **IFTA**  **n, (%)** | 52  (61.9%) | 37  (74.0%) | 15  (44.1%) | 0.006 |
| **Cg>0**  **n,(%)** | 22  (26.2%) | 15  (30,0%) | 7  (20,6%) | 0.427 |
| **Basement membranes multilayering in peritubular capillaries in electron microscopy**  **n, (%)** | 8  (9.5%) | 7  (14.0%) | 1  (2.9%) | 0.393 |

[8] Roufosse C, Simmonds N, Clahsen-van Groningen M*, et al.* A 2018 Reference Guide to the Banff Classification of Renal Allograft Pathology. *Transplantation* 2018; **102:** 1795–1814
